# Supplementary material for: A Semi-Automatic Tool for the Standardized Analysis of Fluorescent Intensity Changes in Polarized Cells
Source: Int J Mol Sci. 2025 Oct 14;26(20):9987. doi: 10.3390/ijms26209987 (PMC12563114; doi:10.3390/ijms26209987)
Supplement: Supplementary file 1 [file ijms-26-09987-s001.zip › Supplementary_detailed_methods.pdf]

Detailed Methods

S.1. Structure and Operation of the Algorithm

Following the import of the TIFF file, each image (frame) is stored as a discrete layer within a list. Consequently, each list element is a matrix representing the pixel intensity values of an individual frame.

S.2. Kneedle

The algorithm requires the input data to be presented in the form of vectors. Specifically, it takes two vectors that together define a set of coordinates – one vector containing the x-values and another containing the corresponding y-values [1]. The subsequent algorithmic process involves the sorting of these data points according to the x-values, followed by the identification of the 'elbow' on the resulting curve (see Figure 9). To achieve this, the system performs a calculation of both the first and second derivatives, subsequently identifying the elbow as the point of maximum curvature.

S.3. Calculation of the Rotation Angle

The program fits a regression line to the image matrix values (creating a scatter plot from the matrix: rows as y coordinate values and columns as x coordinate values of the matrix cells containing an intensity value equal to 1) obtained after 0-1 intensity separation. This separation is a necessary prerequisite for determining the rotation angle, as a suitable regression line can only be fitted to the data format produced by the *image cleaner* output.

S.4. ROI Maker

As a first step, the program locates the matrix elements equal to 1 (demarcating the cell body) within the rotated matrix, the intensities of which have already been converted to a binary form (0 or 1). These values are then exported to a data frame together with their corresponding row and column indices, which can be used to reference them in the original matrix.

The function then plots a curve representing the number of values equal to 1 in each column of the same matrix (Figure 4.). If the process and the soma are distinguishable, there are 2 local maxima on the plot (Figure 4D). On this plot, three lines are also displayed to indicate the number of values of 1 required for the program to recognise the column as part of the cell. This way the size of the ROIs can be ascertained so that they can be adapted to the dimensions of the process.

The positions of the ROIs are determined by selecting columns in which the number of values equal to 1 exceeds the preset threshold from the plot. This approach ensures that the ROIs are exclusively placed on the cell and do not span the gap between the process and the cell body. The program then identifies the rows containing values equal to 1 in the selected columns of the process and calculates the median of the row indices. The ROIs are positioned around this median, with their height spanning the interquartile range of the process's row

indices and their width spanning the width of the process. The upper-lower border is defined as the biggest boxplot's upper and lower quartiles (Figure S1). The same sized ROIs are defined on the soma starting again where the pixel values in the columns reach the threshold, upper-lower borders are defined to be in the same distance from the median in each ROI.

Additionally, the program also leaves a 10-pixel wide gap after the ROI of the process so that the ROIs are indeed placed only on the cell and that there is no ROI in between.

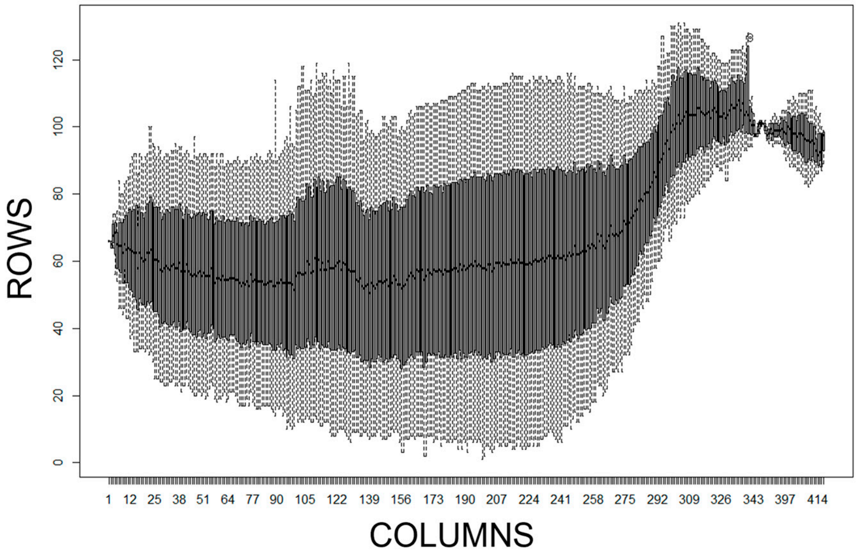

Figure S3. Boxplots of the row indices containing values equal to 1 in each column. Since the row indices are consecutive numbers, the medians are positioned close to the midline of the cell, providing an optimal arrangement to study, for example, intracellular wave propagation of the investigated signal. The interquartile range differs between the soma and the process; therefore, the interquartile range of the process was used to define the height of all ROIs.

S.5. Motion Detection

Using the Wilcoxon signed-rank test, the program quantifies the discrepancy between the median intensity values of the ROIs and those of the background. If a significant difference is found, the shift is not considered a drift. However, if no significant difference is detected between the median intensities of the background ROI and the process ROI, or in a quarter of the ROIs on the cell body, the program will recalculate the ROI positions. In such cases, it returns to the ROI maker function and repeats the steps from that point.

References

1. Tam E. (2022) Kneedle. https://github.com/etam4260/kneedle.
