# Supplementary material for: A Semi-Automatic Tool for the Standardized Analysis of Fluorescent Intensity Changes in Polarized Cells
Source: Int J Mol Sci. 2025 Oct 14;26(20):9987. doi: 10.3390/ijms26209987 (PMC12563114; doi:10.3390/ijms26209987)
Supplement: Supplementary file 1 [file ijms-26-09987-s001.zip › Supplementary_FigureS1.pdf]

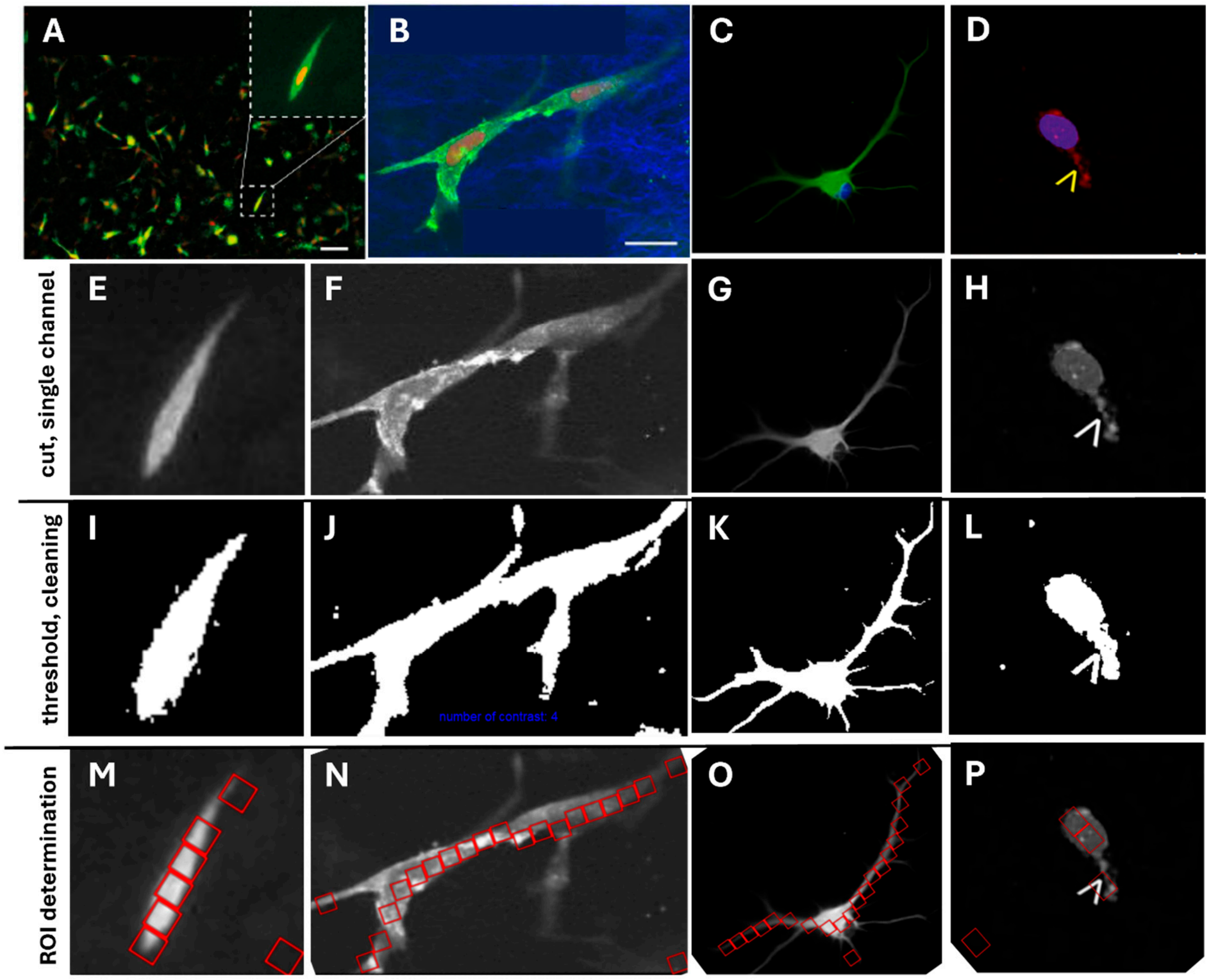

Supplementary Figure S1. ROI determination works with different types of cells and labelling methods. We tested already published fluorescent images of processed cells: A) myoblast C2C12 cell line from Figure 2D of the publication [1] membrane labelled with enhanced green fluorescent protein, B) same study, figure 5A middle inlet, cell with labelled membrane, C) astrocyte from [2] figure 1C labelled for glial fibrillary acidic protein (GFAP) and D) isolated odontoblast from study [3] figure 4B, stained with anti-dentine sialoprotein. We removed or cut the original titles and additional information from the figures. With FIJI, colour channels were split and chosen, the green channel in case of A-C resulted in E-G, and red channel in case of D resulted in H. The saved tiff files were inserted in the program, thresholded and cleaned if needed (I-L) and ROIs were determined (M-P). The number of the ROIs depended mostly on the size of the cell.

References

1. Kasahara K, Muramatsu J, Kurashina Y, Miura S, Miyata S, Onoe H. (2023) Spatiotemporal single-cell tracking analysis in 3D tissues to reveal heterogeneous cellular response to mechanical stimuli. Sci Adv, 9: 1–12.

2. Levy AF, Zayats M, Guerrero-Cazares H, Quiñones-Hinojosa A, Searson PC. (2014) Influence of basement membrane proteins and endothelial cell-derived factors on the

morphology of human fetal-derived astrocytes in 2D. PLoS One, 9: 1–8.

3. Cuffaro HM, Pääkkönen V, Tjäderhane L. (2016) Enzymatic isolation of viable human odontoblasts. Int Endod J, 49: 454–461.
