# Supplementary material for: A Semi-Automatic Tool for the Standardized Analysis of Fluorescent Intensity Changes in Polarized Cells
Source: Int J Mol Sci. 2025 Oct 14;26(20):9987. doi: 10.3390/ijms26209987 (PMC12563114; doi:10.3390/ijms26209987)
Supplement: Supplementary file 1 [file ijms-26-09987-s001.zip › Supplementary_FigureS2.pdf]

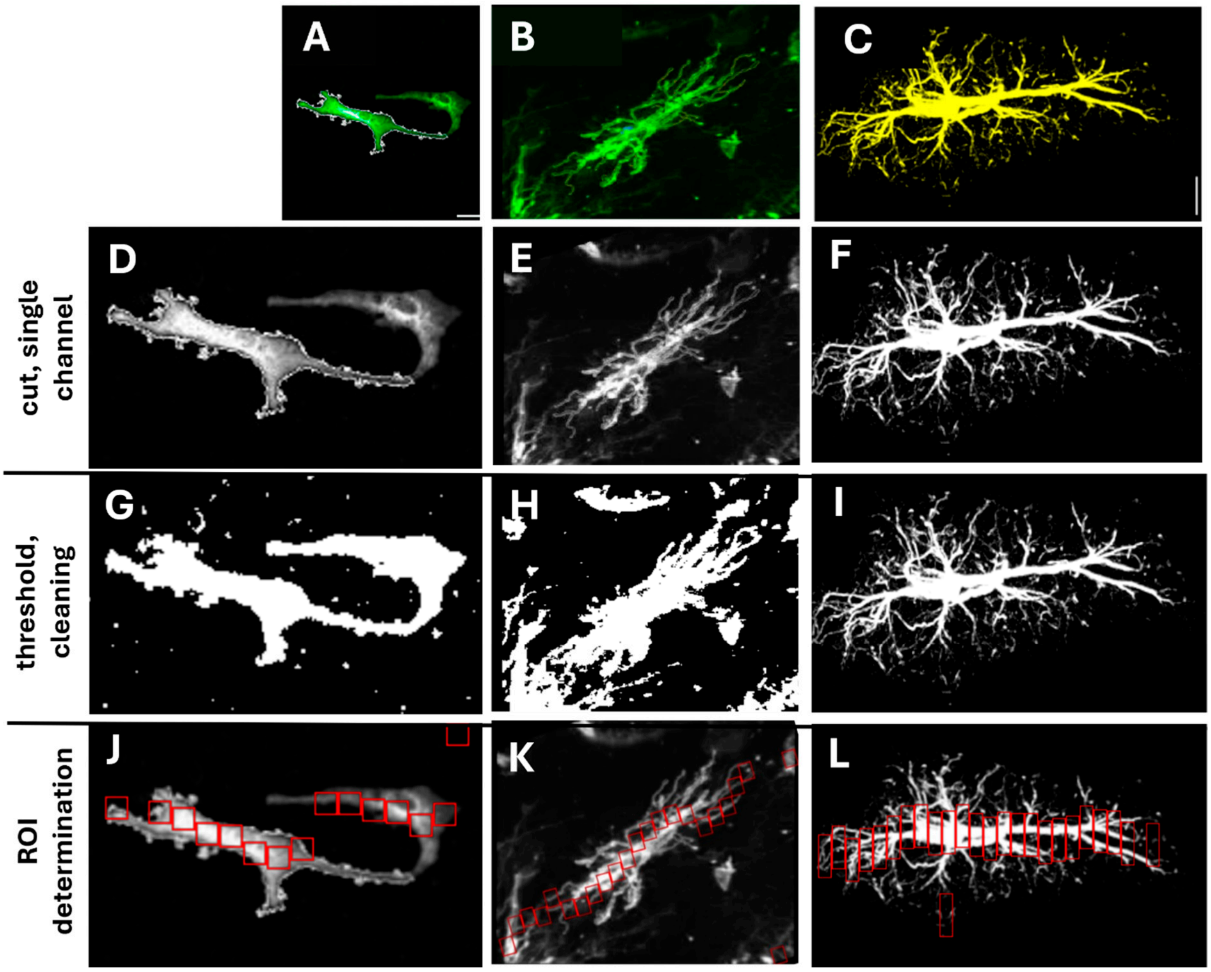

Supplementary Figure S2. ROI determination is not always perfect, but some cases still could be used. We tested already published fluorescent images of processed cells: A) astrocyte from [1] figure 1A, lower left corner image of a cell labelled for RhoA proteins – outlined by the authors, C) same study figure 5B stained for glial fibrillary acidic protein (GFAP) and B) astrocyte from [2] figure 5B labelled for GFAP. We removed or cut the original titles and additional information from the figures. With FIJI, colour channels were split and chosen, the green channel resulted in D-F panels. Saved tiff files were inserted in the program, thresholded and cleaned if needed (G-I) and ROIs were determined (J-L). Because of the complex morphology, the ROIs do not locate on the axis of the cells, however, if the cell is symmetrical (same number of processes are on both sides of the axis) the program define ROIs localized nicely on the cell – depending on the research question these ROI determination can be useful. In panel J) astrocyte's process turns back which forms an overlap in the labelled structures if they are projected to 1 dimension. The program calculates the median of the location in case of thresholded pixels and will determine the location by numerousness. In this cell morphology this program cannot be used reliably at this moment.

References

1. Domingos C, Müller FE, Passlick S, Wachten D, Ponimaskin E, Schwarz MK, Schoch S, Zeug A, Henneberger C. (2023) Induced Remodelling of Astrocytes In Vitro and In Vivo

by Manipulation of Astrocytic RhoA Activity. Cells, 12.

2. Waxman S, Quinn M, Donahue C, Falo LD, Sun D, Jakobs TC, Sigal IA. (2023) Individual astrocyte morphology in the collagenous lamina cribrosa revealed by multicolor DiOlistic labeling. Exp Eye Res, 230 March.
