# Supplementary material for: A Semi-Automatic Tool for the Standardized Analysis of Fluorescent Intensity Changes in Polarized Cells
Source: Int J Mol Sci. 2025 Oct 14;26(20):9987. doi: 10.3390/ijms26209987 (PMC12563114; doi:10.3390/ijms26209987)
Supplement: Supplementary file 1 [file ijms-26-09987-s001.zip › Supplementary_TableS1.pdf]

Supplementary Table S1. Existing laboratory made programs and some features of them. The programs were developed for the analysis of Ca^{2+} imaging video recordings, most of them recommend the use on miniscope level, which involves more tens even hundred cells in the field (mostly neurons, but pancreatic beta cells and myocytes were also involved in the program development). One program mentioned to be prepared for thousands of cell investigation (mesoscale level examination). Automatic ROI determination was solved independently or used previously developed programs. Motion detection is less widespread, only programs made for optogenetic experiment and moving mice it is involved. The opportunity for subcellular investigation was only mentioned in studies but not illustrated. Most of the listed programs are available freely at github.com. However, we must mention that programs which are implemented in MATLAB are not free of charge, because MATLAB is not a free software, even if the codes are open sourced.

ref. no	*program name*	*scale*	*automatic ROI determination*	*motion correction*	*trace extraction*	*subcellular opportunity*	*open source*	*implementation*	*note(s)*
[1]	CITE-On	mesoscale	✓	✓	✓	✗	✓	Python	
[2]	moco	microscope	✓	✓	✓	✗	✓	Java	
[3]	PatchWarp	miniscope	✓	✓	✓	✓ (not illustrated)	✓	MATLAB	focus on the motion correction
[4]	EMC^2	miniscope	✓	✓	✓	✓ (not illustrated)	✓	Java	focus on the motion correction
[5]	EZcalcium	miniscope	use CalmAn	use NoRMCorre	✓	✗	✓	MATLAB	
[6]	-	miniscope	✓	✓	✓	✗	✓	MATLAB	focus on the motion correction
[7]	NoRMCorre	miniscope	✓	✓	✓	✗	✓	Python	focus on the motion correction
[8]	CaPTure	miniscope	✓	✗	✓	✗	✓	MATLAB	

[9]	CaImAn	miniscope	✓	✓	✓	✗	✓	Python, MATLAB
[10]	Begonia	miniscope	✓	✓	✓	✗	✓	MATLAB
[11]	BetaBuddy	miniscope	✓	✗	✓	✗	✓	R, Python
[12]	SpecSeg	miniscope	use NoRMCorre	✗	✓	✓ (not illustrated)	✓	MATLAB
[13]	CardIAP	miniscope	✓	✓	✓	✗	✓	Python
[14]	SIMA	miniscope	✓	✓	✓	✗	✓	Python

References

1. Sità L, Brondi M, Lagomarsino de Leon Roig P, Curreli S, Panniello M, Vecchia D, Fellin T. (2022) A deep-learning approach for online cell identification and trace extraction in functional two-photon calcium imaging. Nat Commun, 13: 1–22.
2. Dubbs A, Guevara J, Yuste R. (2016) moco: Fast motion correction for calcium imaging. Front Neuroinform, 10 FEB: 1–4.
3. Hattori R, Komiyama T. (2022) PatchWarp: Corrections of non-uniform image distortions in two-photon calcium imaging data by patchwork affine transformations. Cell Reports Methods, 2: 100205. doi:10.1016/j.crmeth.2022.100205.
4. Lagache T, Hanson A, Pérez-Ortega JE, Fairhall A, Yuste R. (2021) Tracking calcium dynamics from individual neurons in behaving animals. PLoS Comput Biol, 17: 1–25.
5. Cantu DA, Wang B, Gongwer MW, He CX, Goel A, Suresh A, Kourdougli N, Arroyo ED, Zeiger W, Portera-Cailliau C. (2020) EZcalcium: Open-Source Toolbox for Analysis of Calcium Imaging Data. Front Neural Circuits, 14 May: 1–9.
6. Desai NS, Zhong C, Kim R, Talmage DA, Role LW. (2024) A simple MATLAB toolbox for analyzing calcium imaging data in vitro and in vivo. J Neurosci Methods, 409 April: 110202. doi:10.1016/j.jneumeth.2024.110202.
7. Mitani A, Komiyama T. (2018) Real-time processing of two-photon calcium imaging data including lateral motion artifact correction. Front Neuroinform, 12 December: 1–13.
8. Tippani M, Pattie EA, Davis BA, Nguyen C V., Wang Y, Sripathy SR, Maher BJ, Martinowich K, Jaffe AE, Page SC. (2022) CaPTure: Calcium

PeakToolbox for analysis of in vitro calcium imaging data. BMC Neurosci, 23: 1–14. doi:10.1186/s12868-022-00751-7.
9. Giovannucci A, Friedrich J, Gunn P, Kalfon J, Brown BL, Koay SA, Taxidis J, Najafi F, Gauthier JL, Zhou P, Khakh BS, Tank DW, Chklovskii DB, Pnevmatikakis EA. (2019) Caiman an open source tool for scalable calcium imaging data analysis. Elife, 8: 1–45.
10. Bjørnstad DM, Åbjørsbråten KS, Hennestad E, Cunen C, Hermansen GH, Bojarskaite L, Pettersen KH, Vervaeke K, Enger R. (2021) Begonia—A Two-Photon Imaging Analysis Pipeline for Astrocytic Ca2+ Signals. Front Cell Neurosci, 15 May: 1–12.
11. Alsup AM, Fowlds K, Cho M, Luber JM. (2024) BetaBuddy: An automated end-to-end computer vision pipeline for analysis of calcium fluorescence dynamics in β-cells. PLoS One, 19 3 MARCH: 1–16. doi:10.1371/journal.pone.0299549.
12. de Kraker L, Seignette K, Thamizharasu P, van den Boom BJG, Ferreira Pica I, Willuhn I, Levelt CN, Togt C van der. (2022) SpecSeg is a versatile toolbox that segments neurons and neurites in chronic calcium imaging datasets based on low-frequency cross-spectral power. Cell Reports Methods, 2: 100299. doi:10.1016/j.crmeth.2022.100299.
13. Velez Rueda AJ, Gonano LA, Smith AG, Parisi G, Fornasari MS, Sommese LM. (2023) CardIAP: calcium transients confocal image analysis tool. Front Bioinforma, 3 July: 1–7.
14. Kaifosh P, Zaremba JD, Danielson NB, Losonczy A. (2014) SIMA: Python software for analysis of dynamic fluorescence imaging data. Front Neuroinform, 8 September: 1–10.
