# Supplementary figures and images for: A Semi-Automatic Tool for the Standardized Analysis of Fluorescent Intensity Changes in Polarized Cells
Source: Int J Mol Sci. 2025 Oct 14;26(20):9987. doi: 10.3390/ijms26209987 (PMC12563114; doi:10.3390/ijms26209987)

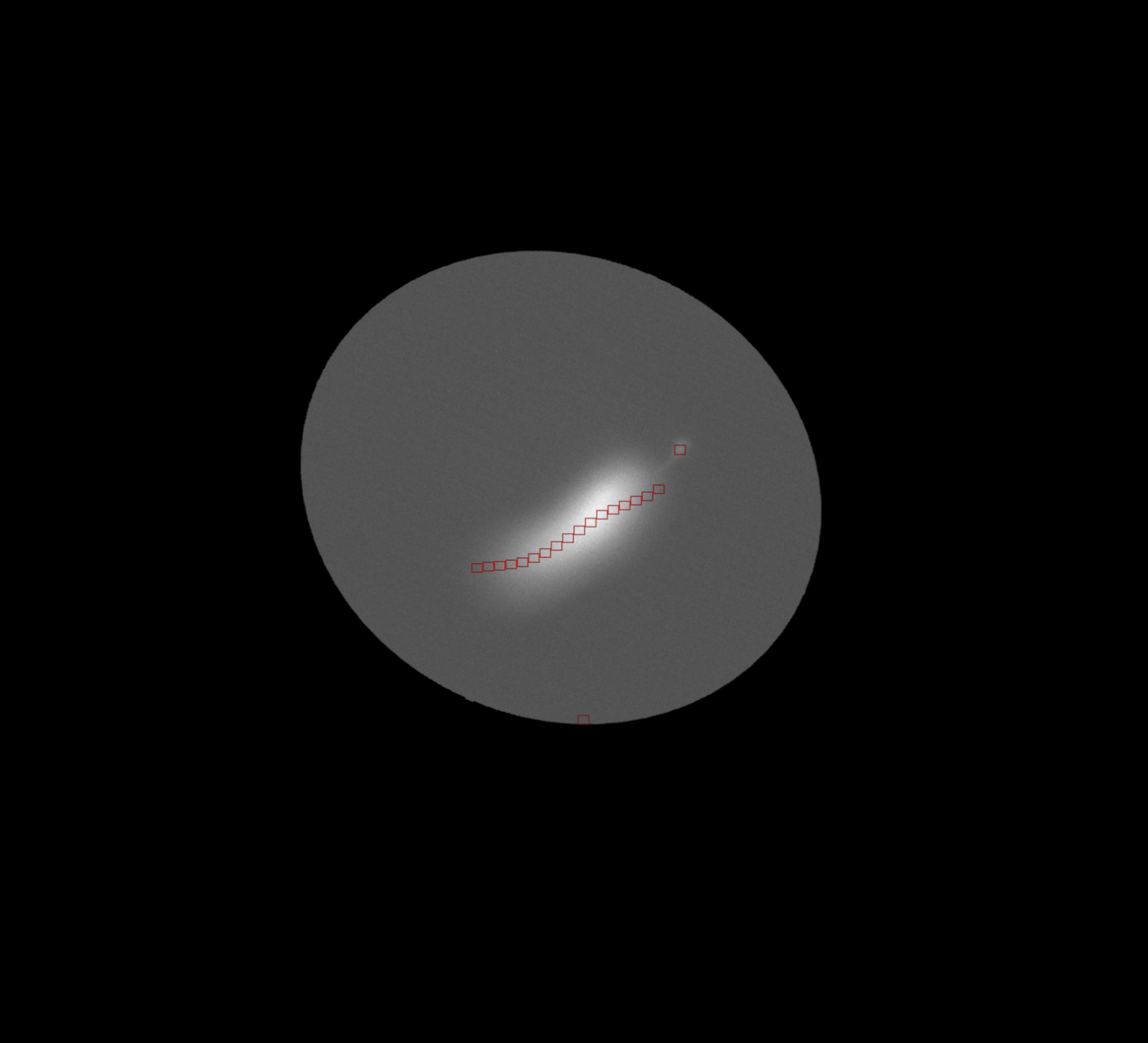

Supplement: Supplementary file 1 [file ijms-26-09987-s001.zip › Example_experiment_17d11001/17d11001_p0001.tif]

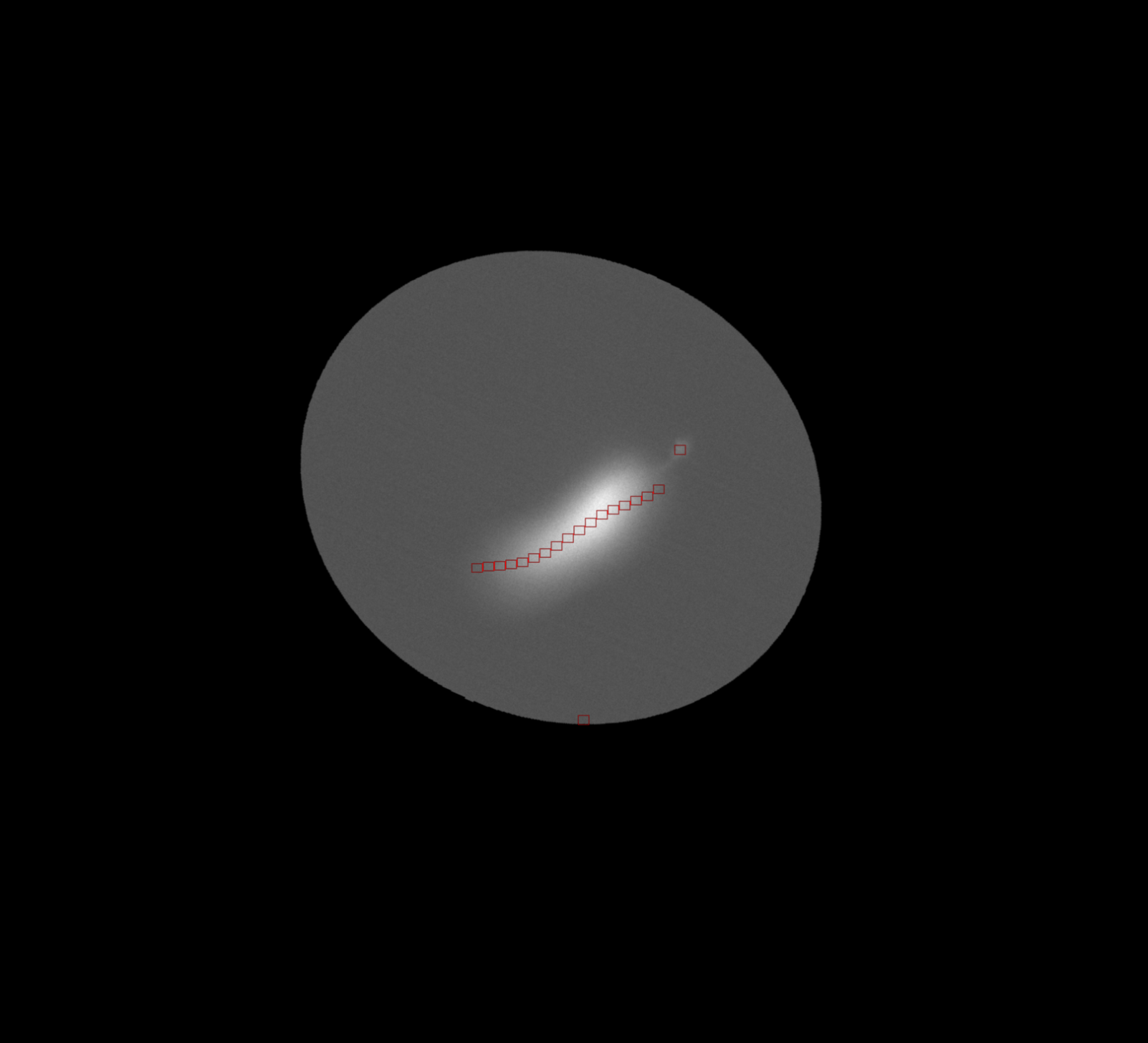

Supplement: Supplementary file 1 [file ijms-26-09987-s001.zip › Example_experiment_17d11001/17d11001_p01.tif]
